# Health behavior changes and mortality among South Korean cancer survivors

**DOI:** 10.1038/s41598-022-20092-z

**Published:** 2022-09-26

**Authors:** Wonjeong Jeong, Eun-Cheol Park, Chung Mo Nam, Sohee Park, Jin Young Nam, Sung-In Jang

**Affiliations:** 1 Cancer Knowledge & Information Center, National Cancer Control Institute, National Cancer Center, Goyang, Republic of Korea; 2 Institute of Health Services Research, Yonsei University, Seoul, Republic of Korea; 3 Department of Preventive Medicine & Institute of Health Services Research, Yonsei University College of Medicine, 50 Yonsei-ro, Seodaemun-gu, Seoul, 03722 Republic of Korea; 4 Department of Biostatistics, Graduate School of Public Health, Yonsei University, Seoul, Republic of Korea; 5 Department of Healthcare Management, Eulji University, Sungnam-si 13135, Gyeonggi-do, Republic of Korea

**Keywords:** Cancer, Health care, Oncology

## Abstract

Considering the rapid growth in the number of cancer survivors, the successful management of their health behaviors requires further attention. However, there are lack of information about cancer survivors’ health behaviors and the risk of mortality using Korean cohort data. This study aimed to examine the effects of health behavior changes on mortality among cancer survivors and to develop a validated nomogram. This cohort study was conducted using claims data. Data from adult cancer survivors from the National Health Insurance Service–National Sample Cohort, conducted between 2002 and 2015, were included. Individuals who were alive for five years after their cancer diagnosis were defined as cancer survivors. Cox proportional-hazards regression was used to estimate the target associations. Discrimination (Harrell’s C-index) and calibration (Hosmer–Lemeshow test) were employed to validate the nomogram. Data from 9300 cancer survivors were used for analysis. Compared to non-smokers, those who started or quit smoking had a higher risk of all-cause mortality. Those who were physically inactive had a higher risk of all-cause mortality than those who were continuously active. In the nomogram, the C-index value was 0.79 in the training data and 0.81 in the testing data. Hosmer–Lemeshow test was not significant, demonstrating a good fit. We found that individuals with unhealthy behaviors had a higher risk of mortality, thereby highlighting the importance of managing health behaviors among cancer survivors. The development of a validated nomogram may provide useful insights regarding official policies and existing practices in healthcare systems, which would benefit cancer survivors. Our study could provide the evidence to inform the priority of guideline for managing the health behavior among cancer survivors.

## Introduction

With improvements in the early detection and treatment of cancer, the long-term outcomes for cancer patients have improved, leading to an increase in the number of cancer survivors^1,2^. These trends of increase in the incidence of cancer and the number of cancer survivors are also seen in Korea^3^. The 5-year relative survival rate of patients with cancer in South Korea improved substantially from 42.9% in 1993–1995 to 70.4% in 2013–2017^4^. As this number is expected to grow dramatically in the next decades, attention is being paid to the health problems faced by cancer survivors^5^.

Concerns about the quality of life of cancer survivors and physical and mental health problems due to neglected healthcare during cancer treatment are increasing^6^. Cancer survivors have many unique healthcare needs, including managing the sequelae of cancer treatment and monitoring for cancer recurrence^7^. Challenges for long-term survivors include psychosocial late effects that call for comprehensive management in which primary care physicians play a critical role^5^. The comprehensive care of cancer survivors must address appropriate health promotion and disease prevention strategies for other conditions, to reduce their physical and psychological distress^8,9^.

Cancer survivors’ health behaviors have important implications for morbidity and mortality^10^. As more people diagnosed with cancer live longer, the implications of lifestyle behaviors such as cigarette smoking that worsen cancer prognosis, are of major importance for public health^11^. Healthy behaviors, such as being physically active, adopting a healthy diet, not smoking, and limiting alcohol intake, may prevent the onset and development of late effects, reduce the incidence of cancer recurrence, and increase life expectancy of cancer survivors^11,12^. However, the prevalence of cigarette smoking among cancer survivors remains high^13,14^. Many cancer survivors continue to smoke despite the knowledge that continued smoking leads to poor clinical outcomes and shorter survival times^13,15^. Interestingly, a study showed that more than 50% of cancer survivors who smoked attempted to quit smoking unsuccessfully^13^. Considering the high proportion of unsuccessful quitting attempts among cancer survivors, there is a need for improvement and integration of smoking cessation measures^13^. Therefore, increasing attention is being paid to such continued health problems to help cancer survivors successfully manage their health behaviors^3^.

It is important to provide support to cancer survivors to help them incorporate healthy behaviors in their lifestyle to improve their long-term health outcomes^16^. Studies have shown that cancer survivors, who had received health information and support to promote physical activity, showed increased physical activity compared to other self-care groups^17^. However, guidelines for helping cancer survivors develop and maintain healthy behaviors in South Korea are still lacking. To systematically manage the health of cancer survivors, a Korean version of the cancer survivor healthcare model is needed.

It is necessary to investigate the effects of health behavior changes and the risk of mortality among cancer survivors to prevent premature mortality by managing their health behaviors. We hypothesized that cancer survivors with healthy behaviors, such as physical activity and not smoking, will have a lower risk of mortality than those with unhealthy behaviors. Consequently, this study examined the risk of mortality according to changes in health behaviors among South Korean cancer survivors. We aimed to develop a validated nomogram to predict the survival rate in cancer survivors.

## Results

Table 1 shows the incidence rates of all-cause mortality among the cancer survivors. Among 9300 cancer survivors, 351 all-cause mortalities were identified during the 245,571.9 person years. Appendix 1 shows the general characteristics of the cancer survivors according to smoking status. Among 9300 cancer survivors, 235 (2.5%) started smoking, 880 (9.5%) continued smoking, 964 (10.4%) quit smoking after their diagnosis, and 7221 (77.6%) were nonsmokers. Appendix 2 shows the general characteristics of the cancer survivors according to physical activity. Among 9300 cancer survivors, 2197 (23.6%) were consistently active, 1915 (20.6%) increased physical activity after diagnosis, 1857 (20.0%) decreased physical activity, and 3331 (35.8%) were physically inactive.

**Table 1:** Incidence rates of all-cause mortality in the cancer survivors’ cohort.

Exposure	Number of participants	Number of deaths	Person years	Incidence rate (95% CI) per 100,000 person years
Total	9300	351	245,571.9	
**Smoking status**				
Started smoking	235	21	5895.9	356.1 (228.1–555.9)
Continued smoking	880	36	23,172.2	155.3 (111.4–216.6)
Quit smoking	964	53	25,022.4	211.8 (160.7–279.1)
Non-smoking	7,221	241	191,481.3	125.8 (110.7–143.1)
**Physical activity**				
Continuously active	2,197	52	58,810.4	88.4 (67.2–116.4)
Increase	1,915	58	50,919.5	113.9 (87.7–147.8)
Decrease	1,857	84	48,667.2	172.6 (138.7–214.7)
Inactive	3,331	157	87,174.8	180.1 (153.5–211.3)

Table 2 shows the effects of health behavior changes on all-cause and cancer-related mortality among cancer survivors. Compared to those who were non-smokers, those who started or quit smoking had a higher risk of all-cause mortality. The risk of cancer-related mortality was not statistically significant. These findings are shown in the results of the risk of mortality among cancer patients within 5 years from diagnosis (Appendix 3), which demonstrate that those who continued smoking or quit smoking had a higher risk of cancer-related mortality than non-smokers before their 5-year survival. Those who were physically inactive had a higher risk of all-cause mortality than those who were continuously active.

**Table 2:** Association between health behavior changes and mortality among cancer survivors.

Variables	All-cause mortality			Cancer-related mortality		
	Adjusted HR	95% CI		Adjusted HR	95% CI	
**Smoking status**						
Started smoking	1.98	(1.25–3.15)		1.55	(0.77–3.11)	
Continued smoking	1.08	(0.75–1.58)		0.71	(0.39–1.29)	
Quit smoking	1.44	(1.05–1.97)		1.52	(1.00–2.29)	
Non-smoking	1.00			1.00		
**Physical activity**						
Continuously active	1.00			1.00		
Increase	1.20	(0.82–1.75)		1.13	(0.70–1.84)	
Decrease	1.46	(1.03–2.07)		1.34	(0.86–2.10)	
Inactive	1.45	(1.05–2.02)		1.08	(0.70–1.66)	
**Sex**						
Male	1.00			1.00		
Female	0.42	(0.32–0.54)		0.40	(0.28–0.57)	
**Age**						
< 50	1.00			1.00		
50–59	2.59	(1.50–4.50)		2.73	(1.39–5.37)	
60–69	5.87	(3.50–9.83)		5.85	(3.08–11.12)	
≥ 70	13.65	(8.06–23.09)		10.67	(5.48–20.80)	
**Income**						
Low	1.04	(0.78–1.39)		0.95	(0.64–1.43)	
Middle	1.03	(0.81–1.31)		0.93	(0.66–1.30)	
High	1.00			1.00		
**Region**						
Metropolitan	1.00			1.00		
City	1.24	(0.92–1.67)		1.42	(0.96–2.12)	
Rural	1.12	(0.86–1.45)		1.13	(0.79–1.62)	
**Medical insurance**						
Insurance (Corporate)	1.00			1.00		
Insurance (Regional)	1.13	(0.90–1.41)		1.16	(0.85–1.58)	
Medical aid	0.83	(0.20–3.42)		0.89	(0.12–6.62)	
**Disability**						
Yes	0.89	(0.62–1.27)		0.67	(0.38–1.19)	
No	1.00			1.00		
**Alcohol consumption**						
0 times	1.00			1.00		
1–2 times a week	0.60	(0.43–0.85)		0.45	(0.27–0.75)	
≥ 3 times a week	0.81	(0.58–1.13)		0.80	(0.51–1.28)	
**BMI**^**a**^						
Underweight	1.67	(1.19–2.34)		1.28	(0.64–2.57)	
Normal	1.00			1.00		
Overweight	0.77	(0.59–1.02)		0.81	(0.55–1.19)	
Obesity	0.77	(0.59–1.00)		0.89	(0.62–1.27)	
**Cancer type**						
Stomach cancer	1.00			1.00		
Colorectal cancer	0.84		(0.55–1.30)	0.81		(0.43–1.51)
Lung cancer	1.17		(0.73–1.88)	1.89		(1.04–3.45)
Liver cancer	1.92		(1.27–2.90)	1.96		(1.09–3.53)
Other cancer	1.01		(0.74–1.39)	1.04		(0.66–1.65)
**Charlson Comorbidity Index (CCI)**						
0	1.00			1.00		
1	1.48		(0.82–2.69)	1.39		(0.70–2.74)
2	1.44		(0.80–2.58)	1.17		(0.59–2.30)
≥ 3	2.17		(1.24–3.79)	1.33		(0.70–2.56)
**Diabetes before cancer**						
Yes	1.25		(0.98–1.59)	1.09		(0.76–1.56)
No	1.00			1.00		
**Hypertension before cancer**						
Yes	0.95		(0.75–1.20)	0.86		(0.63–1.19)
No	1.00			1.00		
**Year of cancer diagnosis**						
2005	1.00			1.00		
2006	0.82		(0.57–1.17)	0.79		(0.47–1.31)
2007	0.66		(0.46–0.95)	0.68		(0.41–1.13)
2008	0.55		(0.38–0.80)	0.70		(0.42–1.15)
2009	0.36		(0.24–0.54)	0.40		(0.23–0.71)
2010	0.14		(0.08–0.25)	0.17		(0.08–0.37)

^a^Body mass index.

**Figure 1:**
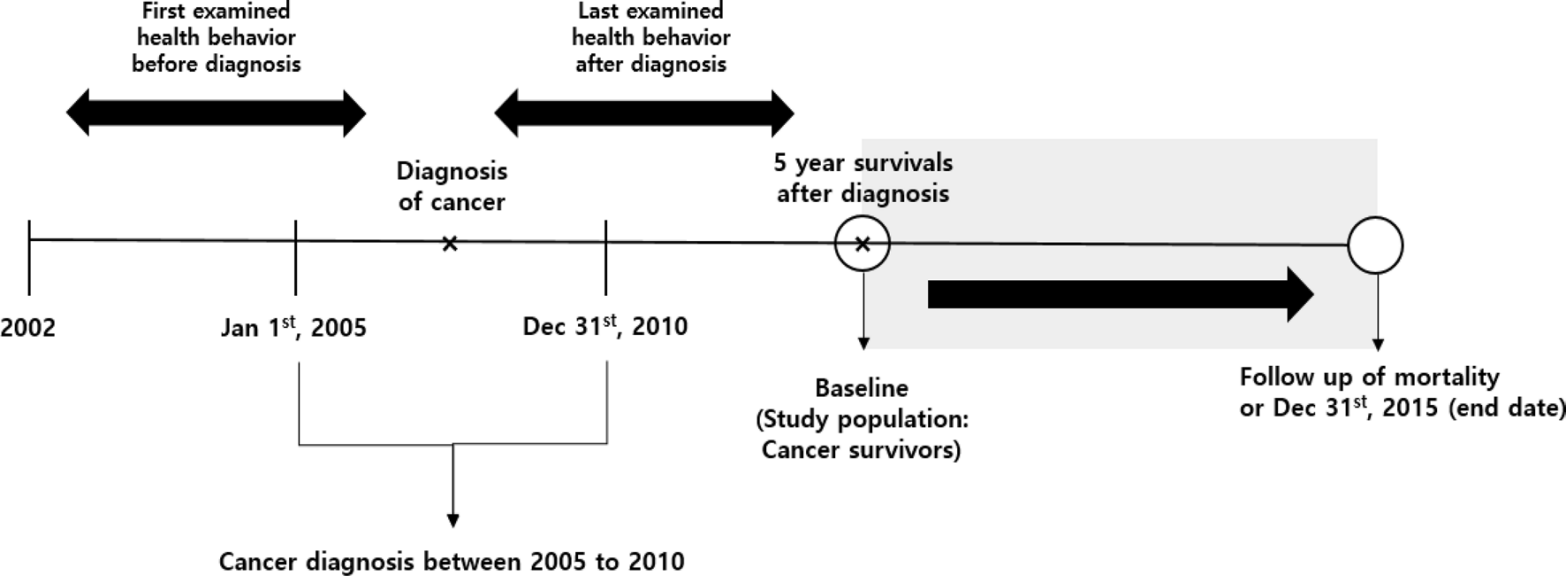
Timeline of the study on health behavior changes among cancer survivors.

Appendix 4 shows the results of subgroup analysis for the risk of all-cause mortality according to smoking status and physical activity changes. Among non-smokers, those who were physically inactive after their cancer diagnosis had a higher risk of all-cause mortality than those who were continuously active (decreased physical activity: HR = 1.56, 95% CI = 1.01–2.40; inactive: HR = 1.68, 95% CI = 1.12–2.53). Appendix 5 shows the results of subgroup analysis for the risk of all-cause mortality and cancer type, according to smoking status and physical activity. Among stomach cancer survivor, those who started smoking had higher risk of all-cause mortality than those who were non-smoking. However, there was no statistically significant results in cancer type according to physical activity (Fig. 1).

**Figure 2:**
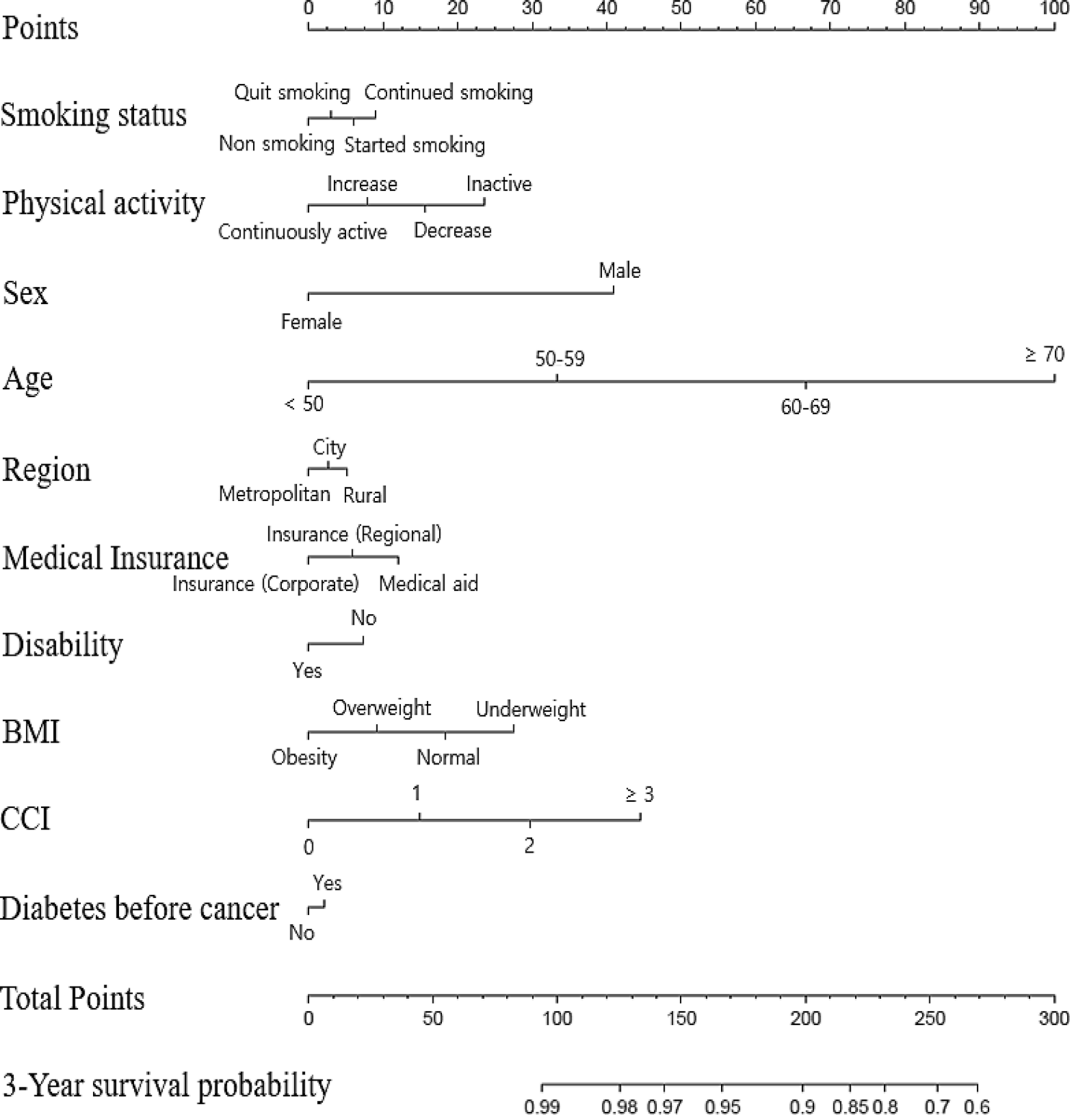
A nomogram for predicting survival in cancer survivors. To develop and validate the nomogram, the variables of smoking status, physical activity, sex, age, region, medical insurance, disability, BMI, Charlson Comorbidity Index, and diabetes before cancer were adjusted.

The 3-year survival nomogram is shown in Fig. 2. The total number of points was calculated by summing the points for each factor. Based on the sum of the assigned number of points for each factor in the nomogram, higher total points were associated with a higher mortality risk. The predicted probabilities of a 3-year survival rate can be obtained by projecting the locations of the total points onto the bottom scales. For example, a male patient who quit smoking, was physically inactive, aged 60–69, living in the city, with medical aid, with no disability, having normal weight, no chronic disease (0 for CCI score), and with diabetes before cancer diagnosis would have a total of 178.5 points (41 points for sex, 3 points for quitting smoking, 23.5 points for physical inactivity, 67 points for age, 2.5 points for region, 12 points for medical insurance, 7.5 points for disability, 20 points for BMI, 0 points for CCI score, and 2 points for diabetes before cancer diagnosis), and had a predicted 3-year survival of 93%.

The C-index value was 0.79 in the training data and 0.81 in the testing data. The C-index is the fraction of all pairs of subjects whose predicted survival times are correctly ordered, which means concordant with actual survival times^18^. A value of 0.79 indicates that 79% of the patients are correctly classified in the training data, considering that C-index is 1.0 indicate a perfect predictive accuracy, and 0.5 indicate the model is not better than random chance^18,19^. The performance of the nomogram was not significantly different between the training and testing datasets. Moreover, the Hosmer–Lemeshow test was not significant (*P* > 0.05), which demonstrated a good fit.

## Discussion

This study investigated the impact of changes in health behavior on the risk of mortality among cancer survivors. First, cox proportional-hazards regression was used to calculate the association between health behavior changes and the risk of mortality among cancer survivors. Our findings indicate that unhealthy behavior is associated with an increased risk of all-cause and cancer-related mortality. Second, the nomogram was constructed based on Cox proportional-hazards regression and validate the model. A validated nomogram was developed to provide information to predict the rate of survival in cancer survivors.

Based on our results, compared to those who were non-smokers, those who started or quit smoking had a higher risk of all-cause mortality. Moreover, those who were physically inactive had a higher risk of all-cause mortality than those who were continuously active. One study shows that cancer survivor’s health behavior plays a crucial role in the posttreatment or tertiary prevention contributing other health outcomes^20^. Exercise is the one of the most important health behaviors for cancer survivors for maintain their health outcomes such as longer survivors, improved disease specific outcomes^21,22^. Smoking is well-known to increase the risk for cancer proliferation and major contributor to cancer mortality^20,23^. Still there are lack of information or guidelines for the survivors to promote their health behaviors, and only 7.6% survivors met all six health behaviors including physical activity, smoking, using sunscreen regularly, alcohol consumption, weight management, and annual primary care visits^24^.

Cancer type is highly associated with their health behavior, such as lung cancer patients were more related to smoking. The distribution of the cancer type might affect the result of the study, but our subjects are similar with the distribution at the targeted period. According to the Korea Central Cancer Registry data, the prevalence of stomach cancer was 18.3%, lung cancer 12.1%, colorectal cancer 12.0%, and liver cancer 10.9% in 2003–2005^25^. Those distributions are similar with the cancer type of our data. The rate of lung cancer was slightly lower, which seems that caused by smoking in the case of lung cancer. These findings demonstrate that those who smoked a lot had higher risk of mortality before their 5-year survival. As our study targeted cancer survivors who survived more than 5 years, the impact of cancer type seems low.

For constructing nomogram, our study divided the study population into training and test group randomly, which was shown in appendix 6. The nomogram was measure using smoking status, physical activity, sex, age, region, medical insurance, disability, BMI, CCI, diabetes before cancer. The model performance was evaluated with respect to discrimination and calibration^26^. The nomogram was subjected to repeatedly extracted resamples about 100 times or more from our original data to obtain C-index values, respectively, and then verified with the average value of the C-index^26^. Harrell’s C-index was performed to examine discrimination and Hosmer–Lemeshow test was used to measure calibration of our model. As all C-indexes in our nomogram were higher than 0.7, the model accuracy means high^27^. This shows that the nomogram performed well in predicting the outcome, which shows similar results with other studies^28^. Calibration was considered good when p-value > 0.05, suggesting that the predicted percentage of survival was in accordance with actual percentage of survival^29^.

Cancer survivors tend to make positive changes in their lifestyles in response to their increased risk of mortality and subsequent health problems, such as with cancer recurrence and other diseases^10^. Research has revealed that cancer diagnosis has been described as a teachable moment for survivors to engage in health-promoting behaviors^30^. Cancer survivors’ health behaviors have important implications for morbidity and mortality, and cancer diagnosis often serves as an impetus for making health behavior changes^10^. Previous studies have shown that young adult survivors have higher levels of life stress, which is related to higher levels of health behavior changes^31^. In one study, cancer survivors even tried to quit smoking as they felt obliged or pressured by healthcare providers, not because they felt ready to quit^32^. Therefore, healthy lifestyle changes, such as increasing physical activity and smoking cessation, are indirectly influenced by physical, emotional, and social adjustment following the diagnosis and treatment that cancer survivors face^30^.

Based on our study, among non-smokers, those who were physically inactive after their cancer diagnosis had a higher risk of all-cause mortality than those who were continuously active. A study showed that compared to those who were in the high-risk unhealthy behavior group (over- or underweight and current or former smokers), those who were in the moderate-risk group (at least two of the three risk factors) had an increased median of 2.1 years, the moderate- to low-risk group (only one of the three risk factors) had an increased median of 3.6 years, and the low-risk group (no risk factors) had an increased median of 5.4 years for their survival^33^. Although modifying one to two behaviors could still lead to a lower risk of mortality, this implies that not participating in any unhealthy behavior is important to obtain the most positive health outcomes.

Physically inactive lifestyles are associated with persistent physical fatigue among cancer survivors for months or even years^34^. One study showed that cancer survivors scored lower on measures of physical performance, suggesting that participation in regular physical activity is not sufficient to overcome the underlying physiological changes imposed by intensified therapies and prolonged hospitalizations^35^. Our study found that those who were physically inactive after diagnosis, especially those whose physical activity decreased, had a higher risk of all-cause mortality than those who were continuously active. Although physical activity is highly associated with cancer survivors’ improved quality of life and positive health outcomes, being physically active after cancer diagnosis is difficult for cancer survivors.

It should be noted that the current study has several limitations. First, the stage or severity of cancer could not be included in this study because the NHIS data were not a cancer registry data, which could not provide the data about cancer severity^36^. However, as we included only cancer survivors who had survived 5 years after diagnosis, the influence of this limitation has decreased and could ensure homogeneity. Additionally, data showed that many lung cancer patients who continuously smoked could not survive for 5 years after their cancer diagnosis. Second, although people with breast cancer are often considered to have a 10-year survival rate, this study could not distinguish breast cancer due to lack of information. The data could not distinguish ICD-10 codes C50–C58 and C60–C63, including breast cancer, as these cancer types were not provided in the NHIS-NSC data^37^. Finally, for the nomogram, only internal validation was performed owing to data limitations, and external validation was not performed; for a more accurate analysis, external validation is necessary. Therefore, caution needs to be taken to interpret other patient cohorts. However, considering that NHIS is the representative data of the South Korean population which is one of the largest data about medical claim, these results might be meaningful. Further researches using larger data sets from different source is necessary.

Despite these limitations, certain strengths of this study are noteworthy. This study used national sampling cohort data to assess the association between changes in health behavior and the risk of mortality. As this data represented the entire South Korean population, it could provide useful information regarding citizens’ utilization of health insurance and health examinations and create higher value-added policies^38^. This study included data collected over a period of nearly 14 years, from 2002 to 2015, and considering that 5-year cancer survivors needed at least 5 years of follow-up, the long follow-up time could enable inferences about a long-term association. Finally, the validated nomogram can provide useful insights into health policies.

The current study identified a significant relationship between changes in health behaviors and mortality among cancer survivors in South Korea. Based on our results, individuals who increasingly engaged in unhealthy behaviors had a higher risk of all-cause and cancer-related mortality. This highlights the importance of managing health behaviors among cancer survivors. We believe that this study can have a meaningful impact not only physically and economically but also socially and politically, by raising awareness about the importance of managing health behaviors among cancer survivors to reduce the risk of adverse health outcomes. Moreover, the development of a validated nomogram may provide useful insights into both official policy and existing practice in healthcare systems, which would benefit further research for cancer survivors.

## Methods

### Data and study participants

Data for this study were obtained from the 2002–2015 National Health Insurance Service–National Sample Cohort (NHIS-NSC). The NHIS-NSC data included all medical claims from approximately 2% of the South Korean population using stratified random sampling. Of these participants, individuals over the age of 20 years were included in this study. We extracted 30,128 individuals who were diagnosed with cancer between 2005 and 2010 using the International Classification of Disease, 10th revision (ICD-10) code: C00-C97. Only 22,629 individuals, who were still alive for 5 years after being diagnosed with cancer, were included and specified as “cancer survivors”^39,40^. Those who were unable to provide information about health behaviors because the data were not collected annually were excluded. Consequently, 9300 individuals were included as cancer survivors. Distribution of target population according to cancer type were shown in Appendix 7, which means that the subjects is similar, although excluding due to lack of information. All data are available in the Korean National Health Insurance Sharing Service database (https://nhiss.nhis.or.kr) and can be accessed upon reasonable request. The NHIS-NSC data are secondary and do not contain any identifying information. This study was reviewed and approved by the Institutional Review Board of Yonsei University Health System (IRB number: 4-2021-1294) and waived the requirement to obtain any informed consent because the data provided by the NHIS were anonymized in compliance with the confidentiality guidelines. This study adhered to the tenets of the Declaration of Helsinki.

### Variables

The risks of all-cause and cancer-related mortality were the dependent variables in this analysis. As only 5-year cancer survivors were included, mortality occurred 5 years after diagnosis. For cancer-related mortality, the death codes were classified according to ICD-10 codes. Among the 5-year cancer survivors, the data were analyzed for mortality within the follow-up period or by the end of the follow-up period (December 31, 2015). Since each cancer survivor had a different cancer diagnosis date, the 5-year survival date was calculated. The follow-up period was considered from the 5-year survival date to the date of death or December 31, 2015.

The primary independent variable was changes in health behavior, including changes in smoking status and physical activity. Health behaviors were calculated by comparing health behaviors before and after cancer diagnosis until the 5-year survival period. For each cancer survivor, the 5-year survival rate was calculated from the date of cancer diagnosis to the 5-year survival mark (Fig. 1). As data were not collected annually, the first response before cancer diagnosis was used as the status of smoking and physical activity before diagnosis. The last response from cancer diagnosis to 5 years after diagnosis was considered as health behavior after diagnosis. For smoking status, individuals who reported being current smokers were placed in the smoking group, and the data for which were observed from before cancer diagnosis and from cancer diagnosis to the 5-year survival mark. For physical activity, those who were physically active at least once a week were categorized into the physically active group, and the data for which were observed from before cancer diagnosis and from cancer diagnosis to the 5-year survival mark. As the questions about physical activity was subdivided into walking, moderate physical activity, and vigorous physical activity after the year 2009, the answer of moderate physical activity was used.

The controls for covariates during both analyses were sex, age, income, region, medical insurance, disability, alcohol consumption, body mass index (BMI)^41^, cancer type, Charlson Comorbidity Index (CCI) score, diabetes before cancer, hypertension before cancer, and year of cancer diagnosis. The cancer types were categorized as stomach cancer (ICD-10: C16), colorectal cancer (ICD-10: C18–C20), lung cancer (ICD-10: C33–C34), liver cancer (ICD-10: C22), and other cancers, indicating the top incidences of cancer in South Korea. As the National Health Insurance Service (NHIS) provides some data excluding information about cancer type, which is considered sensitive information; breast cancer could not be included as a separate category of cancer in this study due to lack of relevant information^42^. The cancer type was chosen based on the first cancer diagnosis for each individual. Alcohol consumption and BMI were calculated based on the response after cancer diagnosis. For diabetes before cancer and hypertension before cancer, diabetes (ICD-10: E10, E11), and hypertension (ICD-10: I10–I15) respectively, were measured using data collected before the date of cancer diagnosis. Cancer was not included in the CCI score, as all individuals were diagnosed with cancer. AIDS/HIV were not included, as the related data were considered sensitive information and were not provided for the sample cohort.

### Statistical analysis

The chi-square test was used to investigate the general characteristics of cancer survivors, which were reported as frequencies and percentages. Cox proportional-hazards regression was used to calculate the association between health behavior changes and the risk of mortality among cancer survivors; it was determined by the adjusted hazard ratio (HR) and 95% confidence interval (CI). Survival time was defined as the number of days from the 5-year survival date (time zero) to the date of death or December 31, 2015, whichever occurred first.

To construct the nomogram, the data were randomly divided into training and testing data at a split ratio of 3:1. The nomogram was constructed based on Cox proportional-hazards regression using training data, and testing data were used to validate the model that predicts survival risk. For the nomogram, we aimed to develop a main model with 10 predictors^43^. To confirm the predictive accuracy of the nomogram, we assessed the nomogram model performance by examining discrimination (Harrell’s C-index) and calibration (Hosmer–Lemeshow test). Harrell’s C-index was used to reflect the predictive accuracy and discrimination ability of each factor and the nomogram, with values near 1 indicating that the risk scores are good at determining accuracy of prediction^44^. The Hosmer–Lemeshow test was conducted to test the calibration of the nomogram, which was used to examine the goodness-of-fit^44^. In this study, the Hosmer–Lemeshow test for the Cox proportional-hazards regression model was used. With a *P*-value > 0.05, it demonstrates a good fit. For the analysis, the R package ‘rms’ and ‘survival’ were used. All data analyses were performed using SAS Enterprise Guide 7.1 (SAS Institute Inc., Cary, NC, USA) and R Studio 1.0.136 software (R Studio Inc., Boston, MA, USA).

### Ethical declaration

This study was reviewed and approved by the Institutional Review Board of Yonsei University Health System (IRB number: 4–2021-1294) and waived the requirement to obtain any informed consent because the data provided by the NHIS were anonymized in compliance with the confidentiality guidelines. This study adhered to the tenets of the Declaration of Helsinki.

## Supplementary Information

Supplementary Information.

## Data Availability

The datasets generated and/or analysed during the current study are available in the Korean National Health Insurance Sharing Service database repository, (https://nhiss.nhis.or.kr). The datasets used and/or analysed during the current study available from the NHIS on reasonable request.
