# Supplementary material for: Health behavior changes and mortality among South Korean cancer survivors
Source: Sci Rep. 2022 Sep 26;12:16011. doi: 10.1038/s41598-022-20092-z (PMC9513084; doi:10.1038/s41598-022-20092-z)

| **Appendix 1. General characteristics of the Cancer survivors according to Smoking status** | | | | | | | | | | | | |
| --- | --- | --- | --- | --- | --- | --- | --- | --- | --- | --- | --- | --- |
| **Variables** | | **Total** | | **Smoking status** | | | | | | | | ***P*-value** |
|  |  |  |  | **Started smoking** | | **Continued smoking** | | **Quit smoking** | | **Non smoking** | |  |
| **Total** | | 9,300 | (100.0) | 235 | (2.5) | 880 | (9.5) | 964 | (10.4) | 7,221 | (77.6) |  |
| **Physical activity** | | |  |  |  |  |  |  |  |  | 0.1706 | |
|  | Continuously active | 2,197 | (23.6) | 48 | (20.4) | 230 | (26.1) | 249 | (25.8) | 1,670 | (23.1) |  |
|  | Increase | 1,915 | (20.6) | 54 | (23.0) | 182 | (20.7) | 190 | (19.7) | 1,489 | (20.6) |  |
|  | Decrease | 1,857 | (20.0) | 47 | (20.0) | 165 | (18.8) | 209 | (21.7) | 1,436 | (19.9) |  |
|  | Inactive | 3,331 | (35.8) | 86 | (36.6) | 303 | (34.4) | 316 | (32.8) | 2,626 | (36.4) |  |
| **Sex** | |  |  |  |  |  |  |  |  |  | <0.0001 | |
|  | Male | 4,556 | (49.0) | 216 | (91.9) | 833 | (94.7) | 880 | (91.3) | 2,627 | (36.4) |  |
|  | Female | 4,744 | (51.0) | 19 | (8.1) | 47 | (5.3) | 84 | (8.7) | 4,594 | (63.6) |  |
| **Age** | |  |  |  |  |  |  |  |  |  | <0.0001 | |
|  | ＜50 | 2,688 | (28.9) | 79 | (33.6) | 351 | (39.9) | 260 | (27.0) | 1,998 | (27.7) |  |
|  | 50-59 | 2,659 | (28.6) | 55 | (23.4) | 235 | (26.7) | 283 | (29.4) | 2,086 | (28.9) |  |
|  | 60-69 | 2,547 | (27.4) | 62 | (26.4) | 213 | (24.2) | 298 | (30.9) | 1,974 | (27.3) |  |
|  | ≥70 | 1,406 | (15.1) | 39 | (16.6) | 81 | (9.2) | 123 | (12.8) | 1,163 | (16.1) |  |
| **Income** | |  |  |  |  |  |  |  |  |  | 0.0003 | |
|  | Low | 1,835 | (19.7) | 46 | (19.6) | 176 | (20.0) | 183 | (19.0) | 1,430 | (19.8) |  |
|  | Middle | 3,189 | (34.3) | 89 | (37.9) | 360 | (40.9) | 325 | (33.7) | 2,415 | (33.4) |  |
|  | High | 4,276 | (46.0) | 100 | (42.6) | 344 | (39.1) | 456 | (47.3) | 3,376 | (46.8) |  |
| **Region** | |  |  |  |  |  |  |  |  |  | 0.0551 | |
|  | Metropolitan | 3,233 | (34.8) | 73 | (31.1) | 287 | (32.6) | 314 | (32.6) | 2,559 | (35.4) |  |
|  | City | 2,298 | (24.7) | 54 | (23.0) | 230 | (26.1) | 269 | (27.9) | 1,745 | (24.2) |  |
|  | Rural | 3,769 | (40.5) | 108 | (46.0) | 363 | (41.3) | 381 | (39.5) | 2,917 | (40.4) |  |
| **Medical Insurance** | |  |  |  |  |  |  |  |  |  | 0.0066 | |
|  | Insurance (Corporate) | 6,520 | (70.1) | 156 | (66.4) | 631 | (71.7) | 720 | (74.7) | 5,013 | (69.4) |  |
|  | Insurance (Regional) | 2,724 | (29.3) | 77 | (32.8) | 243 | (27.6) | 235 | (24.4) | 2,169 | (30.0) |  |
|  | Medical aid | 56 | (0.6) | 2 | (0.9) | 6 | (0.7) | 9 | (0.9) | 39 | (0.5) |  |
| **Disability** | |  |  |  |  |  |  |  |  |  | 0.0052 | |
|  | Yes | 671 | (7.2) | 28 | (11.9) | 70 | (8.0) | 82 | (8.5) | 491 | (6.8) |  |
|  | No | 8,629 | (92.8) | 207 | (88.1) | 810 | (92.0) | 882 | (91.5) | 6,730 | (93.2) |  |
| **Alcohol Consumption** | | |  |  |  |  |  |  |  |  | <0.0001 | |
|  | 0 time | 6,545 | (70.4) | 101 | (43.0) | 305 | (34.7) | 535 | (55.5) | 5,604 | (77.6) |  |
|  | 1-2 times a week | 1,868 | (20.1) | 75 | (31.9) | 337 | (38.3) | 278 | (28.8) | 1,178 | (16.3) |  |
|  | ≥3 times a week | 887 | (9.5) | 59 | (25.1) | 238 | (27.0) | 151 | (15.7) | 439 | (6.1) |  |
| **BMI** | |  |  |  |  |  |  |  |  |  | 0.0049 | |
|  | Underweight | 295 | (3.2) | 5 | (2.1) | 37 | (4.2) | 36 | (3.7) | 217 | (3.0) |  |
|  | Normal | 3,489 | (37.5) | 83 | (35.3) | 350 | (39.8) | 307 | (31.8) | 2,749 | (38.1) |  |
|  | Overweight | 2,430 | (26.1) | 62 | (26.4) | 228 | (25.9) | 279 | (28.9) | 1,861 | (25.8) |  |
|  | Obesity | 3,086 | (33.2) | 85 | (36.2) | 265 | (30.1) | 342 | (35.5) | 2,394 | (33.2) |  |
| **Cancer type** | |  |  |  |  |  |  |  |  |  | <0.0001 | |
|  | Stomach cancer | 1,045 | (11.2) | 29 | (12.3) | 129 | (14.7) | 187 | (19.4) | 700 | (9.7) |  |
|  | Colorectal cancer | 1,030 | (11.1) | 23 | (9.8) | 103 | (11.7) | 135 | (14.0) | 769 | (10.6) |  |
|  | Lung cancer | 488 | (5.2) | 21 | (8.9) | 64 | (7.3) | 75 | (7.8) | 328 | (4.5) |  |
|  | Liver cancer | 826 | (8.9) | 28 | (11.9) | 136 | (15.5) | 95 | (9.9) | 567 | (7.9) |  |
|  | Other cancer | 5,911 | (63.6) | 134 | (57.0) | 448 | (50.9) | 472 | (49.0) | 4,857 | (67.3) |  |
| **Charlson Comorbidity Index (CCI)** | | | |  |  |  |  |  |  |  | 0.4132 | |
|  | 0 | 1,116 | (12.0) | 30 | (12.8) | 108 | (12.3) | 119 | (12.3) | 859 | (11.9) |  |
|  | 1 | 2,222 | (23.9) | 54 | (23.0) | 229 | (26.0) | 203 | (21.1) | 1,736 | (24.0) |  |
|  | 2 | 2,289 | (24.6) | 50 | (21.3) | 209 | (23.8) | 241 | (25.0) | 1,789 | (24.8) |  |
|  | ≥3 | 3,673 | (39.5) | 101 | (43.0) | 334 | (38.0) | 401 | (41.6) | 2,837 | (39.3) |  |
| **Diabetes before cancer** | | |  |  |  |  |  |  |  |  | 0.0128 | |
|  | Yes | 1,791 | (19.3) | 62 | (26.4) | 150 | (17.0) | 179 | (18.6) | 1,400 | (19.4) |  |
|  | No | 7,509 | (80.7) | 173 | (73.6) | 730 | (83.0) | 785 | (81.4) | 5,821 | (80.6) |  |
| **Hypertension before cancer** | | |  |  |  |  |  |  |  |  | <0.0001 | |
|  | Yes | 3,660 | (39.4) | 90 | (38.3) | 263 | (29.9) | 382 | (39.6) | 2,925 | (40.5) |  |
|  | No | 5,640 | (60.6) | 145 | (61.7) | 617 | (70.1) | 582 | (60.4) | 4,296 | (59.5) |  |
| **Year of Cancer diagnosis** | | |  |  |  |  |  |  |  |  | 0.001 | |
|  | 2005 | 791 | (8.5) | 22 | (9.4) | 93 | (10.6) | 75 | (7.8) | 601 | (8.3) |  |
|  | 2006 | 1,539 | (16.5) | 42 | (17.9) | 159 | (18.1) | 139 | (14.4) | 1,199 | (16.6) |  |
|  | 2007 | 1,702 | (18.3) | 51 | (21.7) | 177 | (20.1) | 152 | (15.8) | 1,322 | (18.3) |  |
|  | 2008 | 1,849 | (19.9) | 52 | (22.1) | 167 | (19.0) | 193 | (20.0) | 1,437 | (19.9) |  |
|  | 2009 | 1,800 | (19.4) | 44 | (18.7) | 151 | (17.2) | 202 | (21.0) | 1,403 | (19.4) |  |
|  | 2010 | 1,619 | (17.4) | 24 | (10.2) | 133 | (15.1) | 203 | (21.1) | 1,259 | (17.4) |  |

| **Appendix 2. General characteristics of the Cancer survivor according to Physical activity** | | | | | | | | | | | | |
| --- | --- | --- | --- | --- | --- | --- | --- | --- | --- | --- | --- | --- |
| **Variables** | | **Total** | | **Physical activity** | | | | | | | | ***P*-value** |
|  |  |  |  | **Continuously active** | | **Increase** | | **Decrease** | | **Inactive** | |  |
| **Total** | | 9,300 | (100.0) | 2,197 | (23.6) | 1,915 | (20.6) | 1,857 | (20.0) | 3,331 | (35.8) |  |
| **Smoking status** | |  |  |  |  |  |  |  |  |  | 0.1706 | |
|  | Started smoking | 235 | (2.5) | 48 | (2.2) | 54 | (2.8) | 47 | (2.5) | 86 | (2.6) |  |
|  | Continued smoking | 880 | (9.5) | 230 | (10.5) | 182 | (9.5) | 165 | (8.9) | 303 | (9.1) |  |
|  | Quit smoking | 964 | (10.4) | 249 | (11.3) | 190 | (9.9) | 209 | (11.3) | 316 | (9.5) |  |
|  | Non smoking | 7,221 | (77.6) | 1,670 | (76.0) | 1,489 | (77.8) | 1,436 | (77.3) | 2,626 | (78.8) |  |
| **Sex** | |  |  |  |  |  |  |  |  |  | <0.0001 | |
|  | Male | 4,556 | (49.0) | 1,353 | (61.6) | 881 | (46.0) | 958 | (51.6) | 1,364 | (40.9) |  |
|  | Female | 4,744 | (51.0) | 844 | (38.4) | 1,034 | (54.0) | 899 | (48.4) | 1,967 | (59.1) |  |
| **Age** | |  |  |  |  |  |  |  |  |  | <0.0001 | |
|  | ＜50 | 2,688 | (28.9) | 754 | (34.3) | 653 | (34.1) | 459 | (24.7) | 822 | (24.7) |  |
|  | 50-59 | 2,659 | (28.6) | 700 | (31.9) | 555 | (29.0) | 564 | (30.4) | 840 | (25.2) |  |
|  | 60-69 | 2,547 | (27.4) | 553 | (25.2) | 470 | (24.5) | 548 | (29.5) | 976 | (29.3) |  |
|  | ≥70 | 1,406 | (15.1) | 190 | (8.6) | 237 | (12.4) | 286 | (15.4) | 693 | (20.8) |  |
| **Income** | |  |  |  |  |  |  |  |  |  | <0.0001 | |
|  | Low | 1,835 | (19.7) | 351 | (16.0) | 393 | (20.5) | 381 | (20.5) | 710 | (21.3) |  |
|  | Middle | 3,189 | (34.3) | 692 | (31.5) | 711 | (37.1) | 568 | (30.6) | 1,218 | (36.6) |  |
|  | High | 4,276 | (46.0) | 1,154 | (52.5) | 811 | (42.3) | 908 | (48.9) | 1,403 | (42.1) |  |
| **Region** | |  |  |  |  |  |  |  |  |  | <0.0001 | |
|  | Metropolitan | 3,233 | (34.8) | 874 | (39.8) | 636 | (33.2) | 685 | (36.9) | 1,038 | (31.2) |  |
|  | City | 2,298 | (24.7) | 637 | (29.0) | 502 | (26.2) | 462 | (24.9) | 697 | (20.9) |  |
|  | Rural | 3,769 | (40.5) | 686 | (31.2) | 777 | (40.6) | 710 | (38.2) | 1,596 | (47.9) |  |
| **Medical Insurance** | | |  |  |  |  |  |  |  |  | <0.0001 | |
|  | Insurance (Corporate) | 6,520 | (70.1) | 1,654 | (75.3) | 1,379 | (72.0) | 1,296 | (69.8) | 2,191 | (65.8) |  |
|  | Insurance (Regional) | 2,724 | (29.3) | 538 | (24.5) | 524 | (27.4) | 552 | (29.7) | 1,110 | (33.3) |  |
|  | Medical aid | 56 | (0.6) | 5 | (0.2) | 12 | (0.6) | 9 | (0.5) | 30 | (0.9) |  |
| **Disability** | |  |  |  |  |  |  |  |  |  | <0.0001 | |
|  | Yes | 671 | (7.2) | 114 | (5.2) | 130 | (6.8) | 135 | (7.3) | 292 | (8.8) |  |
|  | No | 8,629 | (92.8) | 2,083 | (94.8) | 1,785 | (93.2) | 1,722 | (92.7) | 3,039 | (91.2) |  |
| **Alcohol Consumption** | | |  |  |  |  |  |  |  |  | <0.0001 | |
|  | 0 time | 6,545 | (70.4) | 1,324 | (60.3) | 1,317 | (68.8) | 1,365 | (73.5) | 2,539 | (76.2) |  |
|  | 1-2 times a week | 1,868 | (20.1) | 626 | (28.5) | 418 | (21.8) | 318 | (17.1) | 506 | (15.2) |  |
|  | ≥3 times a week | 887 | (9.5) | 247 | (11.2) | 180 | (9.4) | 174 | (9.4) | 286 | (8.6) |  |
| **BMI** | |  |  |  |  |  |  |  |  |  | <0.0001 | |
|  | Underweight | 295 | (3.2) | 44 | (2.0) | 70 | (3.7) | 54 | (2.9) | 127 | (3.8) |  |
|  | Normal | 3,489 | (37.5) | 795 | (36.2) | 742 | (38.7) | 645 | (34.7) | 1,307 | (39.2) |  |
|  | Overweight | 2,430 | (26.1) | 616 | (28.0) | 503 | (26.3) | 493 | (26.5) | 818 | (24.6) |  |
|  | Obesity | 3,086 | (33.2) | 742 | (33.8) | 600 | (31.3) | 665 | (35.8) | 1,079 | (32.4) |  |
| **Cancer type** | |  |  |  |  |  |  |  |  |  | 0.0946 | |
|  | Stomach cancer | 1,045 | (11.2) | 259 | (11.8) | 197 | (10.3) | 215 | (11.6) | 374 | (11.2) |  |
|  | Colorectal cancer | 1,030 | (11.1) | 256 | (11.7) | 179 | (9.3) | 209 | (11.3) | 386 | (11.6) |  |
|  | Lung cancer | 488 | (5.2) | 108 | (4.9) | 101 | (5.3) | 92 | (5.0) | 187 | (5.6) |  |
|  | Liver cancer | 826 | (8.9) | 209 | (9.5) | 168 | (8.8) | 180 | (9.7) | 269 | (8.1) |  |
|  | Other cancer | 5,911 | (63.6) | 1,365 | (62.1) | 1,270 | (66.3) | 1,161 | (62.5) | 2,115 | (63.5) |  |
| **Charlson Comorbidity Index (CCI)** | | | | |  |  |  |  |  |  | <0.0001 | |
|  | 0 | 1,116 | (12.0) | 300 | (13.7) | 265 | (13.8) | 200 | (10.8) | 351 | (10.5) |  |
|  | 1 | 2,222 | (23.9) | 590 | (26.9) | 462 | (24.1) | 447 | (24.1) | 723 | (21.7) |  |
|  | 2 | 2,289 | (24.6) | 526 | (23.9) | 493 | (25.7) | 429 | (23.1) | 841 | (25.2) |  |
|  | ≥3 | 3,673 | (39.5) | 781 | (35.5) | 695 | (36.3) | 781 | (42.1) | 1,416 | (42.5) |  |
| **Diabetes before cancer** | | |  |  |  |  |  |  |  |  | <0.0001 | |
|  | Yes | 1,791 | (19.3) | 416 | (18.9) | 301 | (15.7) | 434 | (23.4) | 640 | (19.2) |  |
|  | No | 7,509 | (80.7) | 1,781 | (81.1) | 1,614 | (84.3) | 1,423 | (76.6) | 2,691 | (80.8) |  |
| **Hypertension before cancer** | | | |  |  |  |  |  |  |  | <0.0001 | |
|  | Yes | 3,660 | (39.4) | 765 | (34.8) | 712 | (37.2) | 786 | (42.3) | 1,397 | (41.9) |  |
|  | No | 5,640 | (60.6) | 1,432 | (65.2) | 1,203 | (62.8) | 1,071 | (57.7) | 1,934 | (58.1) |  |
| **Year of Cancer diagnosis** | | | |  |  |  |  |  |  |  | 0.0557 | |
|  | 2005 | 791 | (8.5) | 191 | (8.7) | 149 | (7.8) | 162 | (8.7) | 289 | (8.7) |  |
|  | 2006 | 1,539 | (16.5) | 390 | (17.8) | 286 | (14.9) | 314 | (16.9) | 549 | (16.5) |  |
|  | 2007 | 1,702 | (18.3) | 392 | (17.8) | 358 | (18.7) | 363 | (19.5) | 589 | (17.7) |  |
|  | 2008 | 1,849 | (19.9) | 409 | (18.6) | 363 | (19.0) | 377 | (20.3) | 700 | (21.0) |  |
|  | 2009 | 1,800 | (19.4) | 415 | (18.9) | 388 | (20.3) | 345 | (18.6) | 652 | (19.6) |  |
|  | 2010 | 1,619 | (17.4) | 400 | (18.2) | 371 | (19.4) | 296 | (15.9) | 552 | (16.6) |  |
|  |  |  |  |  |  |  |  |  |  |  |  |  |

| **Appendix 3. Results of association between Health Behaviors and Mortality among Cancer patients before 5 year from diagnosis ^a,b^** | | | | | | | | | | |
| --- | --- | --- | --- | --- | --- | --- | --- | --- | --- | --- |
| **Variables** | | **All-cause Mortality** | | | |  | **Cancer-related Mortality** | | | |
|  |  | **Adjusted HR** | **95% CI** | | |  | **Adjusted HR** | **95% CI** | | |
| **Smoking status** | |  |  |  |  |  |  |  |  |  |
|  | Started smoking | 1.03 | (0.59 | - | 1.81) |  | 0.46 | (0.17 | - | 1.27) |
|  | Continued smoking | 1.22 | (0.87 | - | 1.72) |  | 1.58 | (1.05 | - | 2.37) |
|  | Quit smoking | 1.36 | (1.03 | - | 1.80) |  | 1.65 | (1.18 | - | 2.31) |
|  | Nonsmoking | 1.00 |  |  |  |  | 1.00 |  |  |  |
| **Physical activity** | |  |  |  |  |  |  |  |  |  |
|  | Continuously active | 1.00 |  |  |  |  | 1.00 |  |  |  |
|  | Increase | 1.12 | (0.77 | - | 1.62) |  | 1.08 | (0.70 | - | 1.64) |
|  | Decrease | 1.53 | (1.09 | - | 2.16) |  | 1.28 | (0.86 | - | 1.92) |
|  | Inactive | 1.58 | (1.15 | - | 2.16) |  | 1.36 | (0.95 | - | 1.96) |
| ^a^ Adjusted for sex, age, income, region, medical insurance, disability, alcohol consumption, BMI, cancer type, Charlson Comorbidity Index, diabetes before cancer, hypertension before cancer, and the year of cancer diagnosis. | | | | | | | | | | |
| ^b^ Data were analyzed for the cancer diagnosed to mortality within follow up period or before their 5 year survivals from diagnosis. | | | | | | | | | | |

| **Appendix 4. Subgroup Analysis of the association between Risk of all-cause mortality according to Smoking status and Physical activity change** | | | | | | | | | | | | | | | | | |
| --- | --- | --- | --- | --- | --- | --- | --- | --- | --- | --- | --- | --- | --- | --- | --- | --- | --- |
|  |  | **All-cause mortality** | | | | | | | | | | | | | | | |
|  |  | **Continuously active** |  | **Increase** | | | |  | **Decrease** | | | |  | **Inactive** | | | |
|  |  | **Adjusted**  **HR** |  | **Adjusted**  **HR** | **95% CI** | | |  | **Adjusted**  **HR** | **95% CI** | | |  | **Adjusted**  **HR** | **95% CI** | | |
|  |  |  |  |  | **Lower** |  | **Upper** |  |  | **Lower** |  | **Upper** |  |  | **Lower** |  | **Upper** |
| **Smoking status** | | |  |  |  |  |  |  |  |  |  |  |  |  |  |  |  |
|  | Started smoking | 1.00 |  | 1.96 | (0.21 | - | 18.59) |  | 3.66 | (0.45 | - | 29.60) |  | 3.00 | (0.40 | - | 22.67) |
|  | Continued smoking | 1.00 |  | 1.08 | (0.29 | - | 4.01) |  | 1.99 | (0.64 | - | 6.24) |  | 1.25 | (0.41 | - | 3.84) |
|  | Quit smoking | 1.00 |  | 0.57 | (0.21 | - | 1.51) |  | 0.81 | (0.34 | - | 1.92) |  | 1.18 | (0.55 | - | 2.52) |
|  | Non smoking | 1.00 |  | 1.36 | (0.86 | - | 2.17) |  | 1.56 | (1.01 | - | 2.40) |  | 1.68 | (1.12 | - | 2.53) |
|  |  |  |  |  |  |  |  |  |  |  |  |  |  |  |  |  |  |

| **Appendix 5. Subgroup Analysis of the association between Risk of all-cause mortality and cancer type, according to Smoking status / Physical activity change** | | | | | | | | | | | | | | | | | | | | | | | | | | | | | | | | | | |
| --- | --- | --- | --- | --- | --- | --- | --- | --- | --- | --- | --- | --- | --- | --- | --- | --- | --- | --- | --- | --- | --- | --- | --- | --- | --- | --- | --- | --- | --- | --- | --- | --- | --- | --- |
| **A. Smoking status** | | | | | | | | | | | | | | | | | | | | | | | | | | | | | | | | | | |
|  | |  | | **All-cause mortality** | | | | | | | | | | | | | | | | | | | | | | | | | | | | | | |
|  | |  | | **Started smoking** | | | | | | |  | **Continued smoking** | | | | | | | | |  | **Quit smoking** | | | | | | | | |  | **Non smoking** | | |
|  |  |  | | **Adjusted**  **HR** | **95% CI** | | | | | |  | **Adjusted**  **HR** | | | **95% CI** | | | | | |  | **Adjusted**  **HR** | | | **95% CI** | | | | | |  | **Adjusted**  **HR** | | |
|  |  |  | |  | **Lower** | | |  | **Upper** | |  |  |  |  | **Lower** | | |  | **Upper** | |  |  |  |  | **Lower** | | |  | **Upper** | |  |  |  |  |
| **Cancer type** | | | |  |  | | |  |  | |  |  | | |  | | |  |  | |  |  | | |  | | |  |  | |  |  | | |
|  | | Stomach cancer | | 5.40 | (1.81 | | | - | 16.10) | |  | 1.55 | | | (0.61 | | | - | 3.93) | |  | 1.75 | | | (0.82 | | | - | 3.73) | |  | 1.00 | | |
|  | | Colorectal cancer | | - |  | | |  |  | |  | 1.62 | | | (0.51 | | | - | 5.15) | |  | 1.18 | | | (0.38 | | | - | 3.68) | |  | 1.00 | | |
|  | | Lung cancer | | - |  | | |  |  | |  | 0.30 | | | (0.04 | | | - | 2.63) | |  | 0.68 | | | (0.20 | | | - | 2.37) | |  | 1.00 | | |
|  | | Liver cancer | | 3.04 | (0.75 | | | - | 12.35) | |  | 2.47 | | | (0.91 | | | - | 6.69) | |  | 2.46 | | | (0.92 | | | - | 6.55) | |  | 1.00 | | |
|  | | Other cancer | | 2.23 | (1.23 | | | - | 4.04) | |  | 0.87 | | | (0.50 | | | - | 1.51) | |  | 1.41 | | | (0.91 | | | - | 2.19) | |  | 1.00 | | |
|  | |  | |  |  | | |  |  | |  |  | | |  | | |  |  | |  |  | | |  | | |  |  | |  |  | | |
| **B. Physical activity** | | | | | | | | | | | | | | | | | | | | | | | | | | | | | | | | | | |
|  |  | | **All-cause mortality** | | | | | | | | | | | | | | | | | | | | | | | | | | | | | | | |
|  |  | | **Continuously active** | | |  | **Increase** | | | | | | | | |  | **Decrease** | | | | | | | | |  | **Inactive** | | | | | | | |
|  |  | | **Adjusted**  **HR** | | |  | **Adjusted**  **HR** | | | **95% CI** | | | | | |  | **Adjusted**  **HR** | | | **95% CI** | | | | | |  | **Adjusted**  **HR** | | | **95% CI** | | | | |
|  |  | |  |  |  |  |  |  |  | **Lower** | | |  | **Upper** | |  |  |  |  | **Lower** | | |  | **Upper** | |  |  |  |  | **Lower** | | |  | **Upper** |
| **Cancer type** | | |  | | |  |  | | |  | | |  |  | |  |  | | |  | | |  |  | |  |  | | |  | | |  |  |
|  | Stomach cancer | | 1.00 | | |  | 0.59 | | | (0.19 | | | - | 1.84) | |  | 1.39 | | | (0.58 | | | - | 3.33) | |  | 1.29 | | | (0.58 | | | - | 2.90) |
|  | Colorectal cancer | | 1.00 | | |  | 2.55 | | | (0.73 | | | - | 8.95) | |  | 2.72 | | | (0.84 | | | - | 8.79) | |  | 1.87 | | | (0.57 | | | - | 6.08) |
|  | Lung cancer | | 1.00 | | |  | 0.74 | | | (0.18 | | | - | 2.96) | |  | 0.97 | | | (0.25 | | | - | 3.81) | |  | 0.71 | | | (0.19 | | | - | 2.63) |
|  | Liver cancer | | 1.00 | | |  | 1.36 | | | (0.50 | | | - | 3.70) | |  | 1.13 | | | (0.42 | | | - | 3.01) | |  | 0.99 | | | (0.38 | | | - | 2.55) |
|  | Other cancer | | 1.00 | | |  | 1.20 | | | (0.70 | | | - | 2.04) | |  | 1.58 | | | (0.97 | | | - | 2.58) | |  | 1.69 | | | (1.08 | | | - | 2.66) |
|  |  | |  | | |  |  | | |  | | |  |  | |  |  | | |  | | |  |  | |  |  | | |  | | |  |  |

| **Appendix 6. General characteristics of the study population of test group and training group** | | | | | | | | |
| --- | --- | --- | --- | --- | --- | --- | --- | --- |
| **Variables** | | **Total** | | **Test** | | **Train** | | ***P*-value** |
| **Total** | | 9,300 | (100.0) | 2,322 | (25.0) | 6,978 | (75.0) |  |
| **Smoking status** | |  |  |  |  |  |  | 0.3329 |
|  | Started smoking | 235 | (2.5) | 58 | (2.5) | 177 | (2.5) |  |
|  | Continued smoking | 880 | (9.5) | 220 | (9.5) | 660 | (9.5) |  |
|  | Quit smoking | 964 | (10.4) | 264 | (11.4) | 700 | (10.0) |  |
|  | Non smoking | 7,221 | (77.6) | 1,780 | (76.7) | 5,441 | (78.0) |  |
| **Physical activity** | |  |  |  |  |  |  | 0.9477 |
|  | Continuously active | 2,197 | (23.6) | 553 | (23.8) | 1,644 | (23.6) |  |
|  | Increase | 1,915 | (20.6) | 485 | (20.9) | 1,430 | (20.5) |  |
|  | Decrease | 1,857 | (20.0) | 463 | (19.9) | 1,394 | (20.0) |  |
|  | Inactive | 3,331 | (35.8) | 821 | (35.4) | 2,510 | (36.0) |  |
| **Sex** | |  |  |  |  |  |  | 0.9797 |
|  | Male | 4,556 | (49.0) | 1,137 | (49.0) | 3,419 | (49.0) |  |
|  | Female | 4,744 | (51.0) | 1,185 | (51.0) | 3,559 | (51.0) |  |
| **Age** | |  |  |  |  |  |  | 1.0000 |
|  | ＜50 | 2,688 | (28.9) | 671 | (28.9) | 2,017 | (28.9) |  |
|  | 50-59 | 2,659 | (28.6) | 664 | (28.6) | 1,995 | (28.6) |  |
|  | 60-69 | 2,547 | (27.4) | 636 | (27.4) | 1,911 | (27.4) |  |
|  | ≥70 | 1,406 | (15.1) | 351 | (15.1) | 1,055 | (15.1) |  |
| **Income** | |  |  |  |  |  |  | 0.2456 |
|  | Low | 1,835 | (19.7) | 432 | (18.6) | 1,403 | (20.1) |  |
|  | Middle | 3,189 | (34.3) | 818 | (35.2) | 2,371 | (34.0) |  |
|  | High | 4,276 | (46.0) | 1,072 | (46.2) | 3,204 | (45.9) |  |
| **Region** | |  |  |  |  |  |  | 0.5833 |
|  | Metropolitan | 3,233 | (34.8) | 792 | (34.1) | 2,441 | (35.0) |  |
|  | City | 2,298 | (24.7) | 591 | (25.5) | 1,707 | (24.5) |  |
|  | Rural | 3,769 | (40.5) | 939 | (40.4) | 2,830 | (40.6) |  |
| **Medical Insurance** | |  |  |  |  |  |  | 0.2508 |
|  | Insurance (Corporate) | 6,520 | (70.1) | 1,646 | (70.9) | 4,874 | (69.8) |  |
|  | Insurance (Regional) | 2,724 | (29.3) | 658 | (28.3) | 2,066 | (29.6) |  |
|  | Medical aid | 56 | (0.6) | 18 | (0.8) | 38 | (0.5) |  |
| **Disability** | |  |  |  |  |  |  | 0.6792 |
|  | Yes | 671 | (7.2) | 172 | (7.4) | 499 | (7.2) |  |
|  | No | 8,629 | (92.8) | 2,150 | (92.6) | 6,479 | (92.8) |  |
| **Alcohol Consumption** | | |  |  |  |  |  | 0.0973 |
|  | 0 time | 6,545 | (70.4) | 1,595 | (68.7) | 4,950 | (70.9) |  |
|  | 1-2 times a week | 1,868 | (20.1) | 500 | (21.5) | 1,368 | (19.6) |  |
|  | ≥3 times a week | 887 | (9.5) | 227 | (9.8) | 660 | (9.5) |  |
| **BMI** | |  |  |  |  |  |  | 0.85 |
|  | Underweight | 295 | (3.2) | 75 | (3.2) | 220 | (3.2) |  |
|  | Normal | 3,489 | (37.5) | 862 | (37.1) | 2,627 | (37.6) |  |
|  | Overweight | 2,430 | (26.1) | 598 | (25.8) | 1,832 | (26.3) |  |
|  | Obesity | 3,086 | (33.2) | 787 | (33.9) | 2,299 | (32.9) |  |
| **Cancer type** | |  |  |  |  |  |  | 0.4547 |
|  | Stomach cancer | 1,045 | (11.2) | 260 | (11.2) | 785 | (11.2) |  |
|  | Colorectal cancer | 1,030 | (11.1) | 271 | (11.7) | 759 | (10.9) |  |
|  | Lung cancer | 488 | (5.2) | 109 | (4.7) | 379 | (5.4) |  |
|  | Liver cancer | 826 | (8.9) | 217 | (9.3) | 609 | (8.7) |  |
|  | Other cancer | 5,911 | (63.6) | 1,465 | (63.1) | 4,446 | (63.7) |  |
| **Charlson Comorbidity Index (CCI)** | | | |  |  |  |  | 0.4169 |
|  | 0 | 3,346 | (36.0) | 258 | (11.1) | 858 | (12.3) |  |
|  | 1 | 6,666 | (71.7) | 555 | (23.9) | 1,667 | (23.9) |  |
|  | 2 | 6,904 | (74.2) | 590 | (25.4) | 1,699 | (24.3) |  |
|  | ≥3 | 10,621 | (114.2) | 919 | (39.6) | 2,754 | (39.5) |  |
| **Diabetes before cancer** | | |  |  |  |  |  | 0.4724 |
|  | Yes | 7,509 | (80.7) | 1,863 | (80.2) | 5,646 | (80.9) |  |
|  | No | 1,791 | (19.3) | 459 | (19.8) | 1,332 | (19.1) |  |
| **Hypertension before cancer** | | |  |  |  |  |  | 0.9538 |
|  | Yes | 5,640 | (60.6) | 1,407 | (60.6) | 4,233 | (60.7) |  |
|  | No | 3,660 | (39.4) | 915 | (39.4) | 2,745 | (39.3) |  |
|  |  |  |  |  |  |  |  |  |

| **Appendix 7. Distribution statistics of study population according to cancer type** |
| --- |


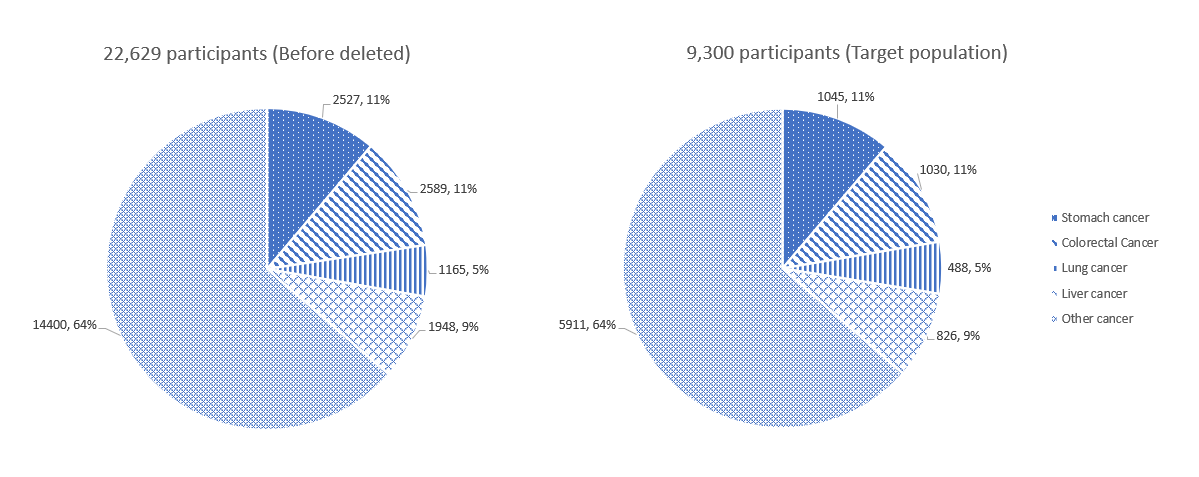

Supplement: Supplementary file 1 — Supplementary Information. [file 41598_2022_20092_MOESM1_ESM.docx]
